# 25-Hydroxycholecalciferol Improves Cardiac Metabolic Adaption, Mitochondrial Biogenetics, and Redox Status to Ameliorate Pathological Remodeling and Functional Failure in Obese Chickens

**DOI:** 10.3390/antiox13111426

**Published:** 2024-11-20

**Authors:** Shih-Kai Chiang, Mei-Ying Sin, Jun-Wen Lin, Maraddin Siregar, Gilmour Valdez, Yu-Hui Chen, Thau Kiong Chung, Rosemary L. Walzem, Lin-Chu Chang, Shuen-Ei Chen

**Affiliations:** 1 Department of Animal Science, National Chung Hsing University, Taichung 40227, Taiwan; g105037017@mail.nchu.edu.tw (S.-K.C.); waterg419@gmail.com (J.-W.L.); d110037008@mail.nchu.edu.tw (M.S.); gilmour18@smail.nchu.edu.tw (G.V.); yuhuey0118@dragon.nchu.edu.tw (Y.-H.C.); 2 Chinese Medicinal Research and Development Center, China Medical University Hospital, Taichung 40447, Taiwan; mysin@dragon.nchu.edu.tw; 3 DSM Nutritional Products Asia Pacific, Mapletree Business City, Singapore 117440, Singapore; thau-kiong.chung@dsm-firmenich.com; 4 Department of Poultry Science, Texas A&M University, College Station, TX 77843, USA; rosemary.walzem@ag.tamu.edu; 5 Center for Molecular Medicine, China Medical University Hospital, Taichung 40402, Taiwan; 6 Department of Biological Science and Technology, China Medical University, Taichung 41354, Taiwan; 7 The iEGG and Animal Biotechnology Center, National Chung Hsing University, Taichung 40227, Taiwan; 8 i-Center for Advanced Science and Technology (iCAST), National Chung Hsing University, Taichung 40227, Taiwan

**Keywords:** hens, vitamin D, heart failure, metabolic adaption, mitochondria, redox homeostasis

## Abstract

Broiler breeder hens allowed ad libitum (Ad) feed intake developed obesity and cardiac pathogenesis and thereby were susceptible to sudden death. A supplement of 69 µg 25-hydroxycholecalciferol (25-OH-D3)/kg feed rescued the livability of feed-restricted (R) and Ad-hens (mortality; 6.7% vs. 8.9% and 31.1% vs. 48.9%). Necropsy with the surviving counterparts along the time course confirmed alleviation of myocardial remodeling and functional failure by 25-OH-D3, as shown by BNP and MHC-β expressions, pathological hypertrophy, and cardiorespiratory responses (*p* < 0.05). 25-OH-D3 mitigated cardiac deficient bioenergetics in Ad-hens by rescuing PGC-1α activation, mitochondrial biogenesis, dynamics, and electron transport chain complex activities, and metabolic adaptions in glucose oxidation, pyruvate/lactate interconversion, TCA cycle, and β-oxidation, as well as in TG and ceramide accumulation to limit lipotoxic development (*p* < 0.05). Supplemental 25-OH-D3 also sustained Nrf2 activation and relieved MDA accumulation, protein carbonylation, and GSH depletion to potentiate cell survival in the failing heart (*p* < 0.05). Parts of the redox amendments were mediated via lessened blood hematocrit and heme metabolism, and improved iron status and related gene regulations (*p* < 0.05). In conclusion, 25-OH-D3 ameliorates cardiac pathological remodeling and functional compromise to rescue the livability of obese hens through metabolic flexibility and mitochondrial bioenergetics, and by operating at antioxidant defense, and heme and iron metabolism, to maintain redox homeostasis and sustain cell viability.

## 1. Introduction

Genetic selection for early rapid growth renders modern broiler chickens susceptible to obesity and related metabolic derangements including ascites and sudden death (SD) [1,2,3,4]. Growth selection in broilers is focused on increasing the mass of breast muscle without corresponding increases in heart and lung capacity that is needed to meet the oxygen requirements of fast-growing muscle tissue [2,5]. The increased workload of the cardiopulmonary system due to altered tissue proportions results in hypertrophic growth of the heart, whereas in the longer term the adaptation often proceeds pathologically, ultimately advancing to functional failure [1,2,5]. While broilers raised for meat are typically harvested at less than 8 weeks of age, breeders that produce the hatching eggs for broiler production live for more than a year, increasing the risk for cardiopulmonary complications and reproductive impairments [3,4]. Very few studies have sought to delineate the relationships between pathogenesis of metabolic dysfunction and SD in adult broilers. Feed restriction to limit muscle growth and adiposity is commonly used throughout the breeder’s lifespan to prevent metabolic derangements and cardiac pathogenesis to improve hens’ livability and egg production [3,4]. This approach, however, has attracted more concerns recently due to chronic animal welfare concerns. Furthermore, due to fighting for food, the distribution of allocated feed is uneven among the flock, despite restricted rations; some hens at a higher hierarchy may receive more portions of feed, leading to obesity development and related disorders.

In humans, cardiomyopathy arising from metabolic disturbance often leads to heart failure [6]. Classically, metabolic cardiomyopathy is characterized by metabolic inflexibility, which gives rise to altered substrate profiles that provoke structural remodeling such as interstitial fibrosis and inflammation, and functional alterations without coronary artery disease, hypertension, or arrhythmias [6]. In most clinical cases, however, heart failure progresses with complications of various systemic dysregulations. We showed that similar metabolic inflexibility and lipotoxic change occur rapidly in broiler breeder hens when released for ad libitum feed intake [3,4].

Our long-standing investigations have shown that heart failure in broiler breeder hens is pathologically similar to that of humans [7,8,9,10]. Bodyweight control by feed restriction greatly limits obesity development and the incidence of heart failure and SD in hens [7,8]. Another similarity extends to the cardioprotective effects of vitamin D supplementation [11,12,13]. The additional inclusion of 69 µg 25-OH-D3/ kg feed greatly relieved systemic dysregulations including hypoxia, acidosis, hypertension, pulmonary artery pressure, vascular remodeling, RAAS (renin–angiotensin–aldosterone system), hyperglycemia, hyperlipidemia, and hepatopathologies such as congestion, fibrosis, and lipid steatosis, as well as myocardial structural remodeling per se including concentric and eccentric hypertrophy, infarction damage, arrhythmia, fibrosis, and inflammation [7,8,9,10]. The faster-growing individuals in both feed-restricted (R-hens) groups and groups of hens allowed sufficient feed for ad libitum consumption (Ad-hens) developed greater adiposity and a higher incidence of cardiac pathologies and SD [8,9]. In contrast to their living counterparts, Ad-hens experiencing SD exhibited anorexia and reduced BW after 8 weeks of free access to feed, while the addition of 69 µg 25-OH-D3/ kg feed delayed the decline of BW for 6 weeks. These results highlight that broiler breeder hens are a good animal model for the investigation of the progression of metabolic cardiomyopathy with systemic dysregulations. The present study aimed to delineate cardiac biogenetics, metabolic adaptations, and redox homeostasis in R-hens and Ad-hens with varying degrees of cardiac remodeling to assess the beneficial effects and underlying mechanisms of 25-OH-D3 on the failing heart.

## 2. Materials and Methods

### 2.1. Animal Management

All bird husbandry and procedures were conducted in accordance with an approved animal care protocol (IACUC Permit NO. 102-113) by The Institutional Animal Care and Use Committee of National Chung Hsing University, Taiwan. A flock of 23-week-old broiler breeder hens (Arbor Acres Plus FF) was purchased from a local breeder farm. Hens were fed to 25 weeks with a nutritionally adequate soy-and-corn-based breeder mash on the weekly adjustment for targeted bodyweights as recommended by the breeder company [3,7]. All birds were caged individually within a house whose ambient temperature was maintained at 26–29 °C. Relative humidity varied with weather and was maintained between 55% and 85%. Birds had free access to water throughout the experiment. Feed was placed at 08:30 a.m. in conjunction with a 14L:10D (lights on at 05:00 a.m.) photo-schedule. At age 26 weeks, 90 birds remained on restricted rations (R-hens) as recommended by the breeder company, while another 90 birds were provided with sufficient feed for consumption to appetite (Ad-hens). Within each feed intake treatment, half of the hens consumed a nutritionally adequate standard breeder diet, while the other half consumed this same diet containing an additional 69 μg/kg feed of 25-OH-D3 (DSM Nutritional Products Ltd., Mapletree Business City, Singapore). Feed formulation was described previously [3,7].

### 2.2. Sampling and Tissue Collection

During the feeding trial, the age of birds experiencing SD was recorded and necropsy completed within 24 h of death. Hearts, livers, and abdominal fat pads were collected for body composition and cardiac hypertrophy analysis. Hearts were deemed hypertrophic when they showed pericardial effusion, ventricular dilation (eccentric hypertrophy), and concentric hypertrophy [3,4,7].

At ages of 28, 33, and 45 weeks (n = 4, 7, and 7, respectively), live hens with BW near their group average were selected for studies. Selected hens were fasted overnight and, the next day, blood pressure and cardiorespiratory response measurements were made in the morning prior to anesthesia and necropsy. Collected organs were used for body composition analysis and pathological examination, as described previously [3,4,7]. Four hearts from each group were used for molecular and biochemical analyses, and the remaining three hearts, collected at 33 and 45 weeks, were used for histochemical and immunostaining studies. For molecular and biochemical studies, left ventricle tissues were minced into 1 mm cubes and washed with PBS thoroughly to remove blood cells. Fresh ventricle tissues were immediately used for analysis or stored under −80 °C until use.

### 2.3. Blood Pressure and Cardiorespiratory Responses

Hens were held in arms until they became placid before blood pressure and heart and respiration rates were measured. Blood pressure measurement was made at the thigh with a digital monitor (Omron Heathcare, Taipei, Taiwan), and heart and respiration rates were measured with a portable monitor (SureSigns VM6; Philips India Limited, Kolkata, India) [7,8,10].

### 2.4. ATP, ADP, Creatine-P Content, Creatine Kinase, and Mitochondrial ETC Complex Activity

Fresh left ventricle tissue was used for ATP and ADP concentration determinations by colorimetric assay according to the instructions of the kits (Cat. #K354-100 and K355-100, respectively, BioVision, Milpitas, CA, USA). Creatine kinase (CrK) activity was measured using a test kit (Cat. #ab155901, Abcam, Cambridge, UK) and creatine phosphate (creatine-P) content was determined by a commercial ELISA kit (Cat. #EK1F699, Biovenic, Hauppauge, NY, USA). Mitochondrial fractions were prepared from fresh left ventricle tissue according to kit protocol (Cat. #ab110168, Abcam) and used for electron transport chain (ETC) complex I, II, and V activity analysis using commercial kits (Cat. #K968-100, K660-100, K2212-100, BioVision, respectively).

### 2.5. TG, Ceramide Content, β-Oxidation, ACC, and Antioxidant Activity

Cardiac triacylglycerol (TG) content was determined following TG separation by thin layer chromatography (TLC) and fatty acid quantification by gas chromatography (GC), and ceramide content was determined by measuring fluorescence of products formed following alkaline hydrolysis, as described previously [14,15]. Approximately, 100 mg of tissue homogenates in assay buffer was used for fatty acid β-oxidation and ACC (acetyl-CoA carboxylase) activity analysis using commercial kits (Cat. #E-141, Biomedical Research Service & Clinical Application, Buffalo, NY, USA, and Cat. #CAK1138, Cohesion Biosciences, London, UK, respectively). Antioxidant activity including superoxide dismutase (SOD) activity and GSH (glutathione) content were measured, and indicators of oxidative damage, malondialdehyde (MDA) indices, and protein carbonylation were determined by commercial kits (Item #706002, 703002, 700870, 10005020, Cayman Chemical Company, Ann Arbor, MI, USA, respectively).

### 2.6. Glycolytic and TCA Cycle Enzyme Activity Analysis

Left ventricle tissue was first homogenized in PBS and centrifuged (10,000× *g* for 10 min at 4 °C), and harvested supernatants were used for lactate content determination using a test kit (Cat. #K607-100, BioVision). Tissue homogenates in the respective assay buffer were used for PDH (pyruvate dehydrogenase), LDH (lactate dehydrogenase), and α-ketoglutarate dehydrogenase (α-KGDH) activity determination using colorimetric kits (Cat. #K679-100, K311-400, K678-100, BioVision, respectively).

Mitochondrial fractions were used for IDH2 (isocitrate dehydrogenase 2, NADP^+^-dependent) and IDH3 (NAD^+^-dependent) activity determination by colorimetric kits (Cat. # K756-100, BioVision). Aliquots of mitochondria fractions were lysed in a buffer (50 mM Tris-HCl, 150 mM NaCl, 2 mM EDTA, 0.2% Triton X-100; *v*/*v*, 0.3% NP-40, protease inhibitor cocktail, pH 7.4), sonicated for 5 × 30 sec bursts with intervening 30 sec rests, and then centrifuged (12,000× *g* at 4 °C for 30 min) prior to supernatant harvest and assay for αKG (α-ketoglutarate) activity measurement with a colorimetric kit (Cat. #K677, BioVision).

### 2.7. Hematocrit, Heme, and Iron Content Analysis

Whole blood samples in heparinized capillary tubes were used for hematocrit measurement. Supernatants from the second centrifugation step of mitochondrial fractionation to pellet the mitochondria (Cat. #ab110168, Abcam) were harvested as the cytosolic fractions. The mitochondrial pellets were then lysed in a buffer (50 mM Tris-HCl, 150 mM NaCl, 2 mM EDTA, 0.2% Triton X-100; *v*/*v*, 0.3% NP-40, protease inhibitor cocktail, pH 7.4), and centrifuged for 10 min at 1000× *g* at 4 °C to remove debris. The heme content of supernatants from lysed mitochondrial and cytosolic fractions was quantified using a colorimetric kit (Cat. #ab272534, Abcam). Total non-heme iron was determined by mixing tissue lysates in acetohydroxamic acid solution (pH 5.2) to release protein-bound iron [16], followed by the procedures enclosed in the kit (K390-100, BioVision).

### 2.8. Histologhy, Immunohistochemistry, and Cell Death Analysis

Paraffin-embedded left ventricle sections were stained by trichrome Masson to visualize tissue fibrosis, as described previously [17]. An antigen retrieval method as described previously was used for immunohistochemical analysis with a mouse anti-chicken MHC-β antibody (clone 2E9, Developmental Studies Hybridoma Bank, Iowa City, IA, USA) to visualize MHC-β expression and an avian-specific mouse monoclonal antibody conjugated with FITC (Clone KUL01, Abcam) for macrophage infiltration [8,17]. Terminal transferase dUTP nick end labeling (TUNEL) assay with de-waxed sections was used for apoptosis analysis (Click-iT^®^ Plus, Roche Applied Science, Indianapolis, IN, USA). Ferroptosis was assessed by Prussian blue staining for iron accumulation [18]. Three sections per hen and five images from each section were used for chromogenic or fluorescence intensity quantification using Image-J software (Version; 1.53, NIH, Bethesda, MD, USA).

### 2.9. Western Blot

Left ventricle homogenates in RIPA buffer, and nuclear extracts prepared using a commercial kit (Cat. #ab113474, Abcam), were used for Western blot analysis using antibodies cross-reactive to chicken antigens including rabbit anti-Nfr-2 (Nuclear factor erythroid 2-related factor 2, Cat. #ab31163) and goat anti-PGC-1α (PPAR-α coactivator 1-alpha, Cat. #ab106814) antibody from Abcam, and rabbit anti-β-actin (Cat. #4967) antibody from Cell Signaling Technology (Danvers, MA, USA), mouse anti-FECH (ferrochelatase, Cat. #sc-377377), HO-1 (heme oxygenase-1, Cat. #sc-390911), Pink1 (PTEN-induced kinase 1, Cat. #sc-518052), and Parkin (Parkinson juvenile disease protein 2, Cat. #sc-32282) antibody from Santa Cruz (Dallas, TX, USA), GPX4 antibody (Cat. #67763-1-Ig) from Proteintech Group, Inc (Rosemont, IL, USA). Horseradish peroxidase-conjugated secondary antibodies (Cell Signaling Technology) were used to identify the bands reactive to the primary antibodies.

### 2.10. Quantification of Mitochondrial DNA

Total DNA were purified using QIAprep Spin Miniprep kits (Qiagen, Hilden, German). Mitochondrial DNA content was determined by the qRT-PCR method using total DNA extracts from whole left ventricle tissues with specific primers targeted to mitochondrial (COX1, CYTB, and ND1) and nuclear (GAPDH) genes (Supplementary Table S1). DNA extracts from mitochondrial fractions were used for actual DNA content quantification using a fluorescent kit (Cat. # ab252898, Abcam). The fluorescence was read at Ex/Em = 492/528 nm.

### 2.11. Gene Expressions by qRT-PCR

Freshly collected left ventricles were quickly dissected into 1 mm cubes on ice, dumped into RNAlaterTM (Invitrogen, Waltham, MA USA), and stored at −80 °C until use. Total RNA extraction was conducted according to the standard procedure enclosed in the Trizol Reagent (Invitrogen, Carlsbad, CA, USA), and 2 μg of total RNA was used for reverse transcription reaction through a random priming method with the use of commercial kits (Applied Biosystems, Waltham, MA, USA). Real-time PCR amplification was performed with commercial SYBR-FAST quantitative PCR kits (Kapa Biosystems, Woburn, MA, USA). Information about the primers is given in Supplementary Table S1. Reactions were conducted in triplicate and the intra-assay CV (coefficient of variation) was less than 10%.

### 2.12. Statistics

Data were analyzed by two-way ANOVA, in which feed intake (Ad or R) and 25-OH-D3 treatment were the classifying variables. Differences between group means were tested using the Bonferroni corrected *t*-test when the main effect was significant. If an interaction between feed intake and 25-OH-D3 treatment was found, a mean comparison was performed. Results are expressed as means ± SE. Mean differences were considered significant at *p* < 0.05. All statistical procedures were carried out using SPSS for Windows 13.0.

## 3. Results

### 3.1. Body Weight, Feed Intake, Mortality, and Cardiac Pathological Hypertrophy in Hens Experiencing SD

Consistent with our previous reports in Cobb breeder hens [7,8], results of body weight, feed intake, heart and abdominal weight, and cardiac pathological hypertrophy in Arbor Acres hens experiencing SD are summarized in Supplementary Materials to avoid repetition and save space (Figures S1A,B,D, S2 and S3A, Table S2). Supplemental 25-OH-D3 significantly improved hens’ livability, with mortality being 8.9%, 6.7%, 48.9%, and 31.1% in R, R+25-OH-D3, Ad, and Ad+25-OH-D3 groups, respectively (*p* = 0.05 in Chi-square analysis, Figure S1C).

### 3.2. Cardiac Pathological Hypertrophy

**Figure 1:**
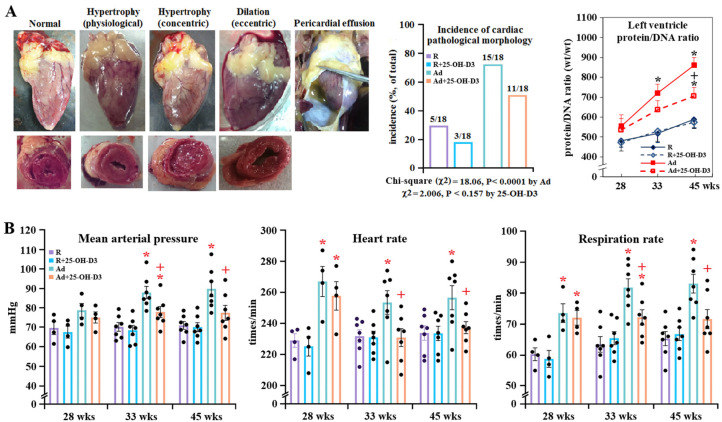
Effects of dietary 25-hydroxycholecalciferol (25-OH-D3) supplementation on cardiac hypertrophy, blood pressure, and cardiorespiratory function of broiler breeder hens provided with restricted (R) or ad libitum (Ad) feed intake. Hens at age of 28, 33, and 45 weeks (n = 4, 7, and 7 from each group, respectively) were necropsied for cardiac remodeling analysis (**A**). Before necropsy, blood pressure, and heart and respiration rates were measured (**B**). Pathological remodeling was judged by concentric hypertrophy, dilation, and pericardial effusion, and results are expressed as an incidence (total n = 18 for each group). *; significant difference by Ad-feed intake (vs. corresponding R-hens, *p* < 0.05). +; significant difference by 25-OH-D3 (vs. R- or Ad-hens, *p* < 0.05).

Release for Ad-feed intake induced cardiac growth and enhanced adiposity as shown by absolute and relative heart and abdominal fat weight and left ventricle protein/DNA ratios along the time course, while supplemental 25-OH-D3 significantly relieved obesity and cardiac hypertrophy in Ad-hens, particularly after 33 weeks (*p* < 0.05, Figure 1A, Figures S2 and S3B). In morphological examination with the hearts collected at 28, 33, and 45 weeks (total n = 18 for each group), most R-hens showed a normal appearance and ventricle wall thickness, and few of them had physiological hypertrophy (noted for a thickened wall but adaptively increased chamber dimensions of the left ventricle), whereas the majority of Ad-hens were characterized by pathological morphologies including concentric hypertrophy (noted for the thickened septum, right and left ventricle wall, and compressed dimensions in the ventricular chamber) and dilation (eccentric hypertrophy, noted for the soft and collapsed myocardium with dramatically enlarged ventricular chamber dimensions), and even in complication with pericardial effusion (noted for fluid accumulation in the space between the pericardium and heart) (Figure 1A). Chi-square analysis showed a significantly increased incidence of the pathologies by Ad-feed intake (*p* < 0.00001) and, to a lower degree, by 25-OH-D3 inclusion (*p*< 0.16). Details of the incidence of respective pathology along the time course are shown in Table S3.

### 3.3. Blood Pressure, Cardiorespiratory Responses, and Myocardial Remodeling

Regardless of feed intake, before death SD-hens exhibited lower heart rates but higher respiratory rates after 29 weeks than their surviving counterparts at the same age, whereas only those of Ad groups showed persistently higher blood pressure [7,8,9]. Accordingly, blood pressure as a peripheral pathological cue and heart and respiratory rates as a cardiorespiratory function were assessed. Ad-feed intake promoted blood pressure and heart and respiratory rates at 28, 33, and/or 45 weeks, while supplemental 25-OH-D3 significantly alleviated blood pressure and heart and respiratory rates at 33 and 45 weeks in Ad-hens (*p* < 0.05, Figure 1B). Since hens were necropsied with similar BW to the average of their respective group, Ad-hens with much higher blood pressures and respiratory rates but lower heart rates therefore may indicate the most susceptible individuals to SD due to a compromised cardiorespiratory system.

**Figure 2:**
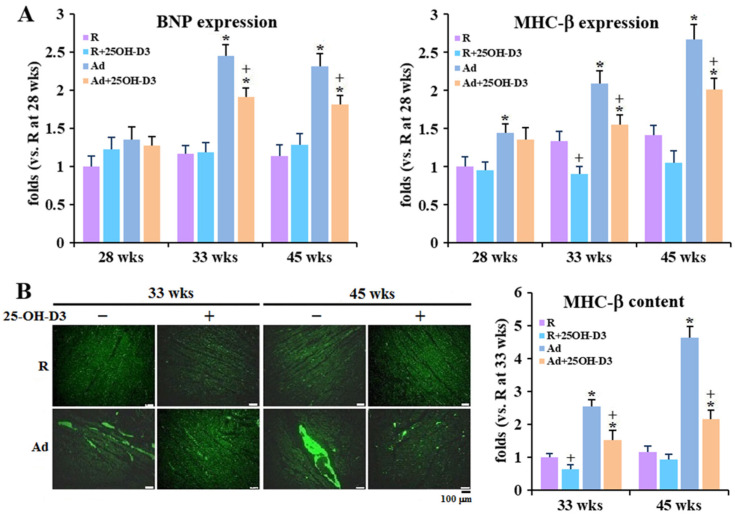
Effects of dietary 25-hydroxycholecalciferol (25-OH-D3) supplementation on heart failure marker expressions of broiler breeder hens provided with restricted (R) or ad libitum (Ad) feed intake. Hearts collected at 28, 33, and 45 weeks were used for cardiac failure marker, BNP (brain natriuretic peptide), and MHC-β (myosin heavy chain, cardiac muscle beta) expressions through the qRT-PCR method with normalization to GADPH (n = 4 (**A**)) and by immunohistochemistry (n = 3 (**B**)). All results are expressed as ratios relative to R-hens at 28 or 33 weeks *; significant difference by Ad-feed intake (vs. corresponding R-hens, *p* < 0.05) +; significant difference by 25-OH-D3 (vs. R- or Ad-hens, *p* < 0.05).

Consistent with relieved cardiorespiratory responses, 25-OH-D3 supplementation significantly ameliorated cardiac fibrosis and inflammation (*p* < 0.05, Figure S4A,B), and attenuated heart failure marker BNP and MHC-β upregulations at 33 and 45 weeks in Ad-hens, and even downregulated MHC-β expressions at 33 weeks in R-hens (*p* < 0.05, Figure 2A,B).

### 3.4. Cardiac Energy Status and Mitochondrial ETC Activity

**Figure 3:**
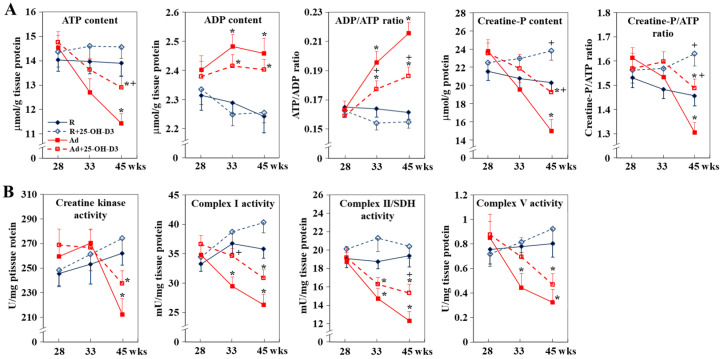
Effects of dietary 25-hydroxycholecalciferol (25-OH-D3) supplementation on cardiac energy status and ETC complex activity of broiler breeder hens provided with restricted (R) or ad libitum (Ad) feed intake. Hearts collected at 28, 33, and 45 weeks were used for ATP and ADP content, CrK (creatine kinase) activity, creatine phosphate (creatine-P) concentration (**A**) determination, and for mitochondrial electron transport chain (ETC) complex I, II, and V activity (**B**) analysis (n = 4) *; significant difference by Ad-feed intake (vs. corresponding R-hens, *p* < 0.05) +; significant difference by 25-OH-D3 (vs. R- or Ad-hens, *p* < 0.05).

In contrast to R-hens, Ad-hens exhibited a decline in cardiac ATP and creatine-P content with significantly lower levels at 45 weeks, but had lower ADP levels at 33 and 45 weeks, and eventually higher ADP/ATP and creatine-P/ATP ratios (*p* < 0.05, Figure 3A). The energy deficiency was associated with impaired CrK activity and mitochondrial oxidative phosphorylation (OXPHOS) as shown by lower ETC complex I (NADH: ubiquinone oxidoreductase), II (succinate-coenzyme Q reductase, also called succinate dehydrogenase; SDH), and V (namely, ATP synthetase) activity at 33 and/or 45 weeks (*p* < 0.05, Figure 3B). CrK responds to increased workload to supply creatine-P for rapid ATP regeneration and downregulation of the CK system reflects energetic starvation of the failing heart [19]. Since Ad-hens had a similar feed intake to those of R-hens at 33 and 45 weeks (Figure S1), the ATP deficiency thus excluded the cause due to insufficient dietary fuels, instead indicating impaired biogenetics in mitochondrial OXPHOS and metabolic inflexibility to replenish the cellular ATP pool. Supplemental 25-OH-D3 significantly alleviated the energy deficit and rescued ETC complex I and II activity at 33 and/or 45 weeks in Ad-hens (*p* < 0.05, Figure 3A,B).

### 3.5. Cardiac Mitochondrial Biogenesis and Dynamics

**Figure 4:**
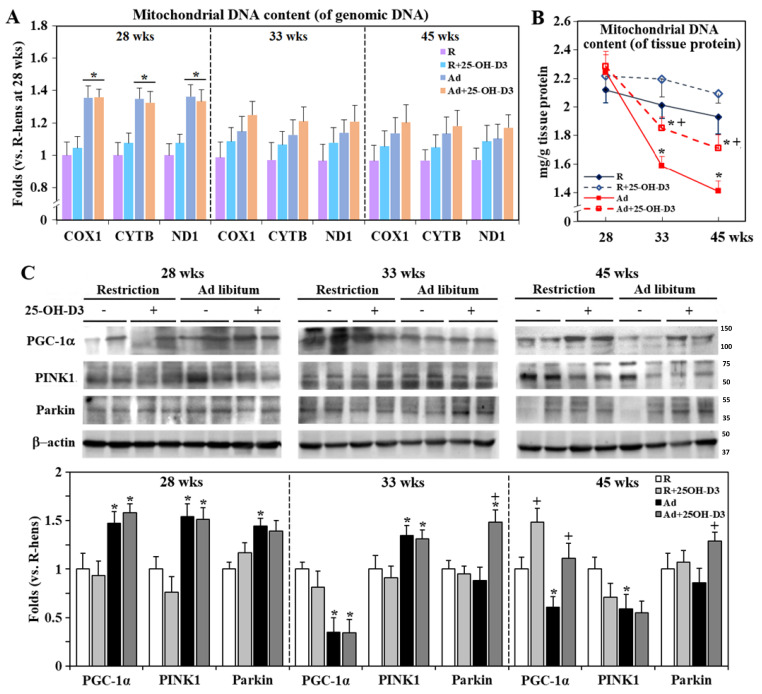
Effects of dietary 25-hydroxycholecalciferol (25-OH-D3) supplementation on cardiac mitochondrial biogenesis and dynamics of broiler breeder hens provided with restricted (R) or ad libitum (Ad) feed intake. Hearts collected at 28, 33, and 45 weeks were used for relative mitochondrial DNA quantification using total DNA extracts for qRT-PCR with specific primers targeted to mitochondrial (COX1, CYTB, and ND1) and nuclear (GAPDH) genes (**A**). Mitochondrial fractions were used for actual DNA content determination using a fluorescence dye method (**B**). Activation of PGC-1α (PPAR-γ coactivator 1-alpha), PINK1 (PTEN-induced kinase 1), and Parkin expressions for mitophagy were analyzed by Western blotting with nuclear extracts and total cell lysates, respectively (**C**). Results of qRT-PCR and Western blot were normalized to GADPH and β-actin, respectively, and expressed as ratios relative R-hens at 28, 33, or 45 weeks (n = 4) *; significant difference by Ad-feed intake (vs. corresponding R-hens, *p* < 0.05) +; significant difference by 25-OH-D3 (vs. R- or Ad-hens, *p* < 0.05). ND1; NADH ubiquinone oxidoreductase chain 1, CYTB; cytochrome b, COX1; cytochrome oxidase 1, GADPH; glyceraldehyde-3-phosphate dehydrogenase.

**Figure 5:**
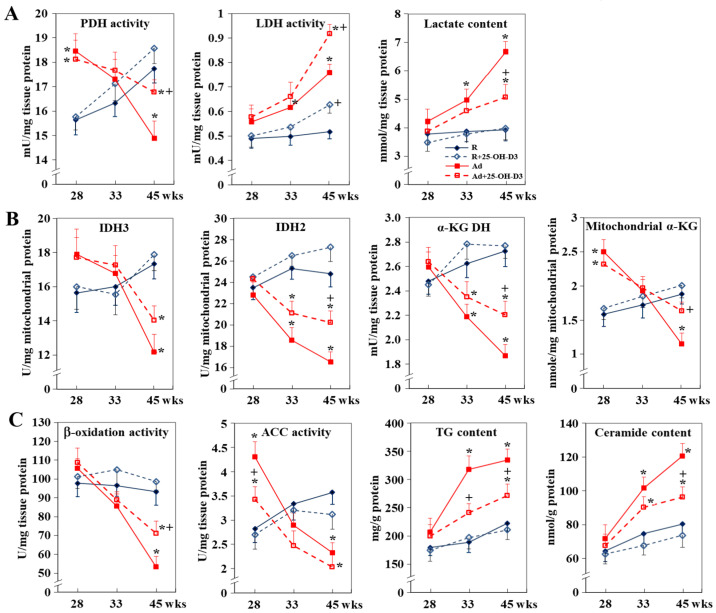
Effects of dietary 25-hydroxycholecalciferol (25-OH-D3) supplementation on cardiac metabolic remodeling of broiler breeder hens provided with restricted (R) or ad libitum (Ad) feed intake. Hearts collected at 28, 33, and 45 weeks were used for PDH (pyruvate dehydrogenase) and LDH (lactate dehydrogenase) activity, lactate concentration (**A**), IDH2 (isocitrate dehydrogenase 2), IDH3, and α-KGDH (α-ketoglutarate dehydrogenase) activity, α-KG concentration (**B**), β-oxidation, ACC activity, TG (triacylglycerol), and ceramide content analysis (**C**) (n = 4) *; significant difference by Ad-feed intake (vs. corresponding R-hens, *p* < 0.05) +; significant difference by 25-OH-D3 (vs. R- or Ad-hens, *p* < 0.05).

Under systemic hypoxia and increased hemodynamic load [8,9], Ad-hens exhibited transiently increased cardiac mitochondrial biogenesis as shown by mitochondrial DNA copies at the basis of nuclear DNA at 28 weeks, but not at the basis of tissue proteins, which further declined to reach significantly lower levels at 33 and 45 weeks (*p* < 0.05, Figure 4A,B). The change was concomitantly associated with transient upregulation of nuclear PGC-1α at 28 weeks, but a decline after 33 weeks (*p* < 0.05, Figure 5C). Supplemental 25-OH-D3 rescued mitochondrial biogenesis at the basis of tissue proteins and PGC-1α activation in Ad-hens, and even upregulated PGC-1α in R-hens at 45 weeks (*p* < 0.05, Figure 4C).

Ad-feed intake upregulated PINK1 and Parkin expressions at 28 and/or 33 weeks but downregulated PINK1 at 45 weeks, while 25-OH-D3 upregulated Parkin at 33 and 45 weeks in Ad-hens (*p* < 0.05, Figure 4C). Since the PINK1/Parkin pathway functions in mitochondrial quality control by targeting impaired mitochondria for degradation (mitophagy) and regulating the fission and fusion process, these results suggest that 25-OH-D3 rescues the mitochondria dynamics in the failing hearts of Ad-hens [20].

### 3.6. Cardiac Metabolic Remodeling

Ad-feed intake promoted cardiac glycolysis as shown by PDH activity for glucose oxidation at 28 weeks, and LDH activity and lactate content at 33 and 45 weeks (*p* < 0.05, Figure 5A), suggesting metabolic adaptions in both aerobic and anaerobic glycolysis to increased cardiac workload. 25-OH-D3 attenuated the drop in PDH activity and potentiated the increase in LDH activity, but reduced lactate accumulation at 45 weeks in Ad-hens (*p* < 0.05, Figure 5A). Since LDH catalyzes the interconversion between pyruvate and lactate, the uncoupled LDH activity and lactate levels under upregulated PDH activity at 45 weeks suggest that 25-OH-D3 shifts the equilibrium toward pyruvate, possibly for oxidation to rescue biogenetics in the failing heart [21,22].

In contrast to R-hens, Ad-hens showed a decline in enzyme activities in the TCA cycle, including IDH3 (NAD^+^-dependent), IDH2 (NADP^+^-dependent), and α-KGDH, with significantly lower levels at 33 and/or 45 weeks, while mitochondrial α-KG, the product of IDH, was accumulated at 28 weeks but downregulated at 45 weeks (*p* < 0.05, Figure 5B). Downregulation of IDH2 and α-KGDH activity reflects mitochondrial oxidative stress in early cardiac hypertrophy [23,24,25]. An impaired TCA cycle thus not only results in insufficient anaplerotic intermediates and electron supply for ETC of OXPHOS biogenetics, but reflects a broad catastrophe in mitochondrial integrity to drive the heart into failure [20]. Supplemental 25-OH-D3 rescued IDH2, α-KGDH activity, and α-KG level at 45 weeks in Ad-hens (*p* < 0.05, Figure 5B).

In contrast to R-hens, Ad-hens exhibited a transient increase in ACC activity at 28 weeks, but together with β-oxidation, declined to significantly lower levels at 45 weeks, while significant TG and ceramide accumulation were observed at 33 and 45 weeks in Ad-hens (*p* < 0.05, Figure 5C), a hallmark of lipotoxicity [26]. 25-OH-D3 rescued β-oxidation and attenuated TG and ceramide accumulation at 33 and/or 45 weeks in Ad-hens (*p* < 0.05, Figure 5C). The increased ACC activity coupled with rapid accumulation of TG and ceramide during 28 to 33 weeks may reflect enhanced lipid synthesis from the de novo pathway due to a burst of feed intake (Figure S1), while the steady increase in TG and ceramide content from 33 to 45 weeks under a decline in ACC activity can be attributed to suppressed β-oxidation and persistently elevated circulating NEFA (non-esterified fatty acids) and TG levels [10].

### 3.7. Cardiac Antioxidant Defense

**Figure 6:**
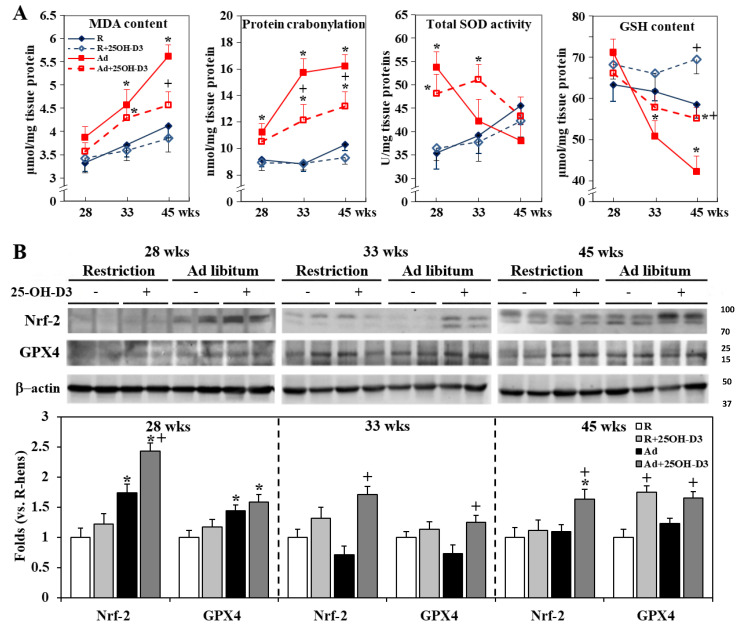
Effects of dietary 25-hydroxycholecalciferol (25-OH-D3) supplementation on cardiac antioxidative capacity of broiler breeder hens provided with restricted (R) or ad libitum (Ad) feed intake. Hearts collected at 28, 33, and 45 weeks were used for MDA (malondialdehyde) and GSH (glutathione) content, protein carbonylation, and SOD (superoxide dismutase) activity analysis (**A**). Nuclear extracts and total tissue lysates were used for Nfr-2 (nuclear factor erythroid 2-related factor 2) activation and GPX4 (glutathione peroxidase 4) expression by Western blotting, respectively (**B**). Results of Western blot were normalized to β-actin and are expressed as ratios relative to R-hens at 28, 33, or 45 weeks (n = 4) *; significant difference by Ad-feed intake (vs. corresponding R-hens, *p* < 0.05) +; significant difference by 25-OH-D3 (vs. R- or Ad-hens, *p* < 0.05).

Ad-feed intake promoted cardiac MDA content and protein carbonylation at 28, 33, and/or 45 weeks, and depleted the GSH reservoir at 33 and 45 weeks, while it transiently upregulated SOD activity, Nrf-2 activation, and downstream target gene GPX4 expression at 28 weeks (*p* < 0.05, Figure 6A,B). 25-OH-D3 ameliorated MDA accumulation, protein carbonylation, and GSH depletion, and upregulated Nrf2 and GPX4 at 28, 33, and/or 45 weeks in Ad-hens, and even GSH content and GPX4 expression at 45 weeks in R-hens (*p* < 0.05, Figure 6A,B).

### 3.8. Cardiac Heme Metabolism and Cell Ferroptosis and Apoptosis

**Figure 7:**
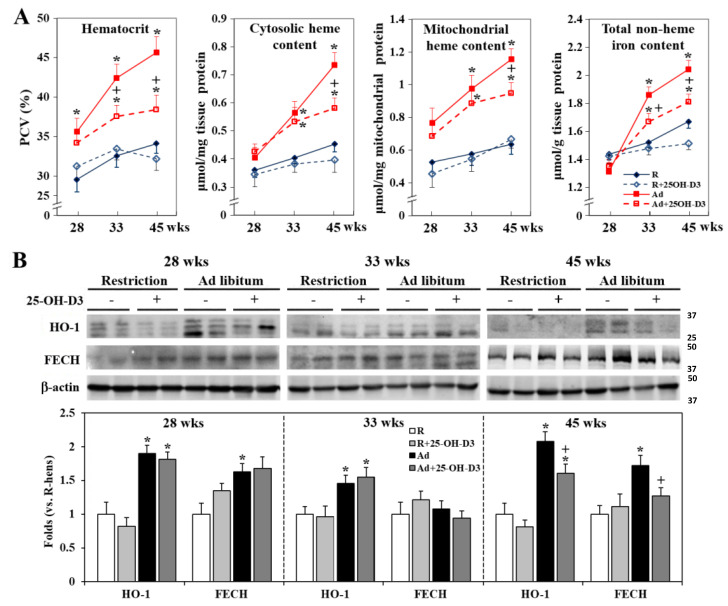
Effects of dietary 25-hydroxycholecalciferol (25-OH-D3) supplementation on hematocrit and cardiac heme metabolism of broiler breeder hens provided with restricted (R) or ad libitum (Ad) feed intake. Blood samples collected at 28, 33, and 45 weeks (n = 4, 7, and 7 from each group) were used for hematocrit analysis and, after necropsy, hearts were used for cytosolic and mitochondrial heme and iron content analysis (n = 4) (**A**). HO-1 (heme oxygenase-1) and FECH (ferrochelatase) expressions were analyzed by Western blotting (**B**). Results of Western blot are normalized to β-actin and expressed as ratios relative to R-hens at 28, 33, or 45 weeks (n = 4) *; significant difference by Ad-feed intake (vs. corresponding R-hens, *p* < 0.05) +; significant difference by 25-OH-D3 (vs. R- or Ad-hens, *p* < 0.05).

Ad-feed intake elevated blood hematocrit values and cardiac cytosolic and mitochondrial heme content, and promoted iron accumulation along the time course, accompanied by upregulated expressions of HO-1 and FECH, the key enzymes in heme degradation and biosynthesis, respectively [27,28] (*p* < 0.05, Figure 7A,B). Since Ad-hens suffered chronic systemic hypoxia [8], these results reflect enhanced heme uptake, catabolism, and synthesis in the failing heart under elevated hematocrit by erythropoiesis [29,30]. Supplemental 25-OH-D3 significantly lowered hematocrit levels, lessened cardiac heme metabolism and iron accumulation, and relieved upregulation of HO-1 and FECH at 33 and/or 45 weeks in Ad-hens (*p* < 0.05, Figure 7A,B).

**Figure 8:**
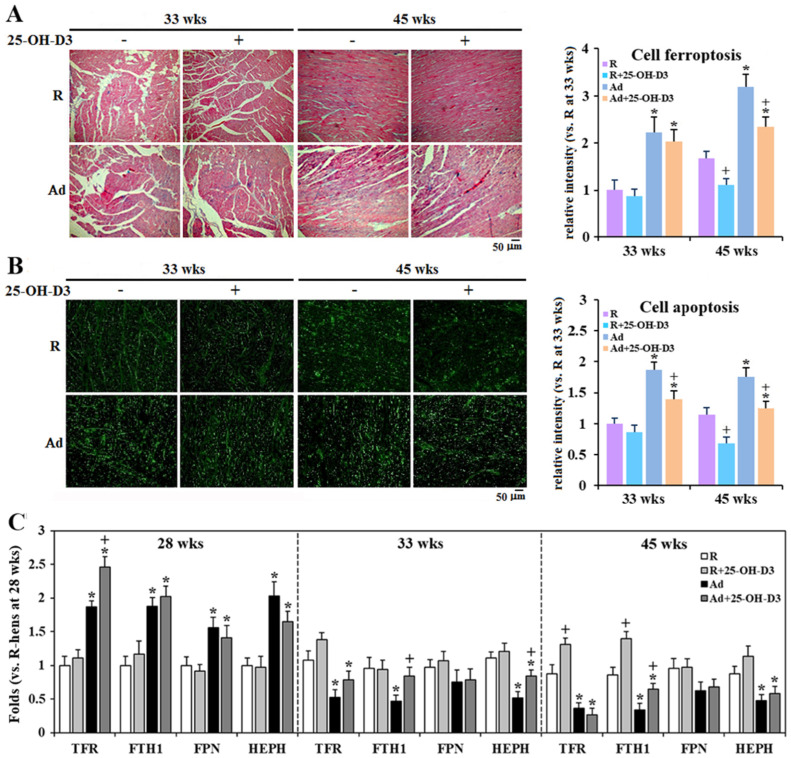
Effects of dietary 25-hydroxycholecalciferol (25-OH-D3) supplementation on cardiac cell death and iron-related gene expressions of broiler breeder hens provided with restricted (R) or ad libitum (Ad) feed intake. Hearts collected at 28, 33, and 45 weeks were used for ferroptosis and apoptosis analysis by Prussian blue staining and TUNEL (terminal deoxynucleotidyl transferase dUTP nick end labeling) assay, respectively ((**A**,**B**), n = 3). Iron-related gene expressions including TFR (Transferrin receptor), FTH1 (Ferritin, heavy chain 1), FPN (Ferroportin-1), and HEPH (Hephaestin) were analyzed by qRT-PCR with normalization to GADPH ((**C**), n = 4). Results are expressed as ratios relative to R-hens at 28 or 33 wks *; significant difference by Ad-feed intake (vs. R-hens at 33 weeks, *p* < 0.05) +; significant difference by 25-OH-D3 (vs. R- or Ad-hens, *p* < 0.05).

Consistent with redox dysregulations and cellular heme and iron accumulation (Figure 7 and Figure 8), Ad-hens showed very pronounced cardiac cell ferroptosis and apoptosis as indicated by Prussian staining and TUNEL assay, respectively (*p* < 0.05, Figure 8A,B) [18,28]. Cardiac iron uptake and disposal in Ad-hens was programmed with compensatory upregulation of related gene expressions, including TFR (Transferrin receptor), FTH1 (Ferritin, heavy chain 1), FPN (Ferroportin-1), and HEPH (Hephaestin) at 28 weeks, but progressed with downregulation of TFR, FTH1, and HEPH at 33 and 45 weeks, leading to labile iron accumulation and, eventually, ferroptotic cell death (*p* < 0.05, Figure 7A and Figure 8C). Supplemental 25-OH-D3 significantly relieved cardiac cell death in both Ad- and R-hens at 33 and/or 45 weeks consistently with rescued or potentiated TFR, FTH1, and HEPH expressions (*p* < 0.05, Figure 8A–C).

## 4. Discussion

In various animal models, vitamin D has been shown to ameliorate myocardial remodeling, including interstitial fibrosis, MHC-β, and ANP (atrial natriuretic peptide) expression, inflammatory status, and metabolic cardiomyopathy [13,26]. Activation of VDR (vitamin D receptor) by 1α, 25-dihydroxycholecalciferol (1,25-(OH)2-D3) downregulates ANP gene transcription by binding to and silencing its promoter activity [31]. In rat cardiomyocytes under endothelin-induced hypertrophy, 1,25-(OH)2-D3 suppressed ANP secretion and total MHC synthesis, and thus reduced cell growth [32]. In addition to systemic hypoxia, hypertension, and type-2 diabetic derangements [8,9,10], the present study further demonstrated that 25-OH-D3 relieves heart failure through structural and metabolic remodeling in the myocardium. In healthy adult men, sympathetic activity produced by paced breathing was shown to impair the correlation between body fat percentage and sympathovagal balance, suggesting that high body fat percentage is associated with a low sympathetic activity in heart rate modulation [33]. In addition to myocardial remodeling, therefore, parts of the altered cardiorespiratory function in Ad-hens may be attributed to enhanced adiposity and chronic hypoxia.

In normal adult hearts, mitochondrial OXPHOS contributes to the majority of ATP production and less than 10% is provided by the net ATP yield in anaerobic glycolysis [34,35]. Regardless of etiologies, a failing heart is characterized by energy starvation with a 30-40% deficit in ATP status and the creatine-P/ATP ratio [19,36]. This starving state is mainly caused by mitochondrial dysfunction as a result of ROS (reactive oxygen species) insults and impaired mitochondrial OXPHOS, biogenesis, and dynamics [20]. The dysregulated cardiac CrK system, including creatine level, CrK activity, and creatine-P/ATP ratio, has been explored as a predictor of mortality in patients with dilated cardiomyopathy [36]. Mitochondrial creatine kinase (CrKMT), which mediates the transfer of high-energy phosphate to cytosolic creatine, is a target gene of VDR [37]. Mice overexpressing M-CrK (the muscle form) even escaped from pressure-induced heart failure [38]. For the first time, the present obese chicken model was also characterized with a severe ATP deficit and the dysfunctional cardiac CrK system in the failing heart as manifested by impaired mitochondrial biogenesis, dynamics, and ETC complex activities, while 25-OH-D3 supplement amended the defects to improve cardiac biogenetics.

The most pivotal cardioprotective effect of vitamin D may rely on the antioxidant system. Both Klotho and Nrf-2 transcription, the two key genes in redox homeostasis, are driven by VDR, which subsequently primes a variety of gene expressions such as catalase, SOD, and various enzymes in GSH and the thioredoxin system [39]. Moreover, PGC-1α interacts with VDR to jointly co-activate VDRE (VDR response element)-harboring promoters for target gene expressions [40]. PGC-1α, as a master coactivator in transcription, responds to remodeling stimuli such as calcium, adrenoreceptor-cAMP signaling, nitric oxide, fibrotic interleukin 4 (IL-4), AMPK (AMP activated protein kinase), Sirtuin, and redox status to potentiate cardiac bioenergetics by interacting with Nrf-1 and -2, PPARα-RAR (peroxisome proliferator-activated receptor α-retinoid X receptor) complex, and ERRα (estrogen-related receptor-α) to prime gene expressions involved in redox regulations, mitochondrial OXPHOS, and fatty acid uptake and metabolism [20,41,42]. Previously, we reported persistent activation of VDR by 25-OH-D3 with cardiac remodeling in Ad-hens [10]. Since blood pressure and cardiorespiratory responses, as well as heart hypertrophy and adiposity, were not altered at 28 weeks, the early upregulation of PGC-1α and Nrf-2 by Ad-feed intake reflects an adaptive response for physiological remodeling independent of energy status and 25-OH-D3 effects. As systemic hypoxia and hypertension occurred and lasted, increased workload imposed the remodeling into pathological development, as shown by persistently augmented HIF-1α activation, calcineurin-NAFT (nuclear factor of activated T-cells) signaling, inflammation, and fibrosis [8,10], as well as by upregulation of ANP, MHC-β, and oxidative stress, in the present study. These pathologies ultimately drove the heart into overt failure, featuring impaired biogenetics and downregulation of PGC-1α during 33 to 45 weeks, while 25-OH-D3 differentially upregulated PGC-1α and Nrf-2 activation and relieved structural and metabolic remodeling during the progression of failure. In PGC-1α knockout mice, the fetal genes of cardiac ANP and BNP and hexokinase 2 were upregulated, whereas the adult form of MHC-6 and PDH kinase 4, a PDH inhibitor, were downregulated, accompanied by damaged contractility and a shift in fuel reliance toward glucose metabolism [42,43,44].

At the late stage of heart failure, most enzyme activities of the TCA cycle, such as IDH, aconitase, and α-KGDH, as well as ETC complexes, are impaired, not simply through gene expressions, but due to protein modifications by specific oxidative addictions [20,25]. Patients at the late stage of heart failure exhibited lower activities of mitochondrial ETC complexes in the left ventricle, particularly those containing iron–sulfur and/or heme redox centers, which were attributed to protein carbonylation by oxidative insults rather than a loss of protein abundance [45]. In addition to the complex I [20], impaired SDH (complex II) instead of complex III and IV was shown as another major site of electron leakage contributing to a lower mitochondrial GSH reservoir in growing broilers with ascites [46]. Inactivated IDH2 by HNE (4-hydroxy-2-nonenal) addiction was observed at the early hypertrophy stage of heart failure [23,24]. Since IDH2 also produces NADPH for GSH/GSSG and the thioredoxin system to maintain redox homeostasis within the mitochondria [47], impaired IDH2 activity therefore was suggested as an early mitochondrial oxidative stress marker in heart failure [23,24]. Moreover, α-KGDH acts not only as a target but also a generator of ROS and its catalysis to yield NADH, which is selectively shuttled to NNT (nicotinamide nucleotide transhydrogenase) for mitochondrial NADPH production to prevent oxidative stress in the heart [25,48]. Accordingly, the VDR-Nrf2 axis and VDR/PGC-1α co-activation in redox regulation of TCA cycle enzymes and ETC complexes mediate the rescued biogenetics by 25-OH-D3 in the failing hearts of Ad-hens. The robust accumulation of mitochondrial α-KG at 28 weeks without alterations in IDH, α-KGDH, and SDH activity may reflect adaptive metabolic remodeling due to an influx of cytosolic α-KG deriving from IDH1 reaction and/or glutaminolysis, as well as from the metabolites of alanine, aspartate, or sugars to replenish intermediates for the functional TCA cycle and mitochondrial biogenetics [49]. Supplemental α-KG in drinking water has been shown to relieve pressure-induced myocardial remodeling in mice [50].

A healthy heart is metabolically flexible in its fuel utilization to sustain contractile function. Fatty acid β-oxidation normally accounts for up to 60% of mitochondrial ATP production in the postnatal heart, followed by glucose oxidation via pyruvate/PDH, then ketones and lactate oxidation to pyruvate, and anaerobic glycolysis, while branched chain amino acids contribute a very minor portion [35,51]. Hearts respond to pressure overload to increase glucose metabolism [52,53], but as the remodeling progresses into an overt failure state, they lose flexibility in fuel utilization [35]. Depending on models, stimuli, and complications and severity in clinical cases, most studies concluded that a failing heart increases anaerobic glycolysis and ketone body oxidation, and decreases or remains unchanged in β-oxidation, while glucose oxidation may be decreased or upregulated, and lactate oxidation is downregulated [20,34,35]. The discrepancy in glucose oxidation can be attributed to the relative abundance of adult and fetal MHC isoforms during the development of heart failure [54]. The robust ACC and PDH activity at 28 weeks without significant changes in IDH, α-KGDH, and SDH activity, and β-oxidation suggest an enhanced non-canonical TCA cycle [55], in which the resultant citrate is shuttled to the cytosol, where citrate is cleaved by ATP citrate lyase to produce acetyl-CoA for palmitate and following lipid synthesis, while another product, oxaloacetate, is converted to malate, which is shuttled back to mitochondria to join the anaplerotic TCA cycle. In contrast to their parents, crossbred mice with transgenic diacylglycerol acyltransferase 1 (DGAT1) and VDR gene knockout showed more pronounced lipotoxic cardiomyopathy [26]. Therefore, supplemental 25-OH-D3 improves metabolic flexibility in glucose oxidation and β-oxidation to potentiate biogenetics and diminish lipotoxic development during cardiac remodeling in Ad-hens.

Recently, several studies highlighted the importance of pyruvate/lactate interconversion and lactate shuttling in modulating cardiac remodeling. Mice with cardio-specific deletion of LDHA, the M-type (muscle) subunit that forms a home- or hetero-tetramer of functional LDH, were characterized with defective cardiac hypertrophy and functional failure under pressure overload [21]. Knockout of MPC (mitochondrial pyruvate carrier for pyruvate import into mitochondria) in adult mouse hearts resulted in hypertrophic pathologies and functional failure, while pharmacological inhibition of MCT4 (lactate exporter monocarboxylate transporter 4) sustained mitochondrial pyruvate flux, relieved ROS production, and prevented and even reversed cardiomyocyte hypertrophy [22]. In addition to functioning as a fuel, lactate stabilizes NDRG3 (N-myc downregulated gene family 3) through physical interaction to mediate hypertrophic signaling [21]. Several clinical studies even confirmed lactate levels as a critical indicator in the prognosis of cardiovascular diseases [56,57]. Lactate released from muscle and other tissues serves as the major cardiac energy source in young men under exercise, even exceeding those from fatty acids [58]. Despite not being defined with lactate origins, the uncoupled lactate level and LDH activity under upregulated PDH at 45 weeks reflect an operative pyruvate/lactate interconversion by 25-OH-D3 for pyruvate production and subsequent oxidation to sustain the TCA cycle in the failing heart [21,22].

Heme exposure has been shown to modify contractile proteins and impair their contractility in cardiomyocytes [59]. In hemolytic mice, heme accumulation in the heart provoked ROS production, altered Ca^2+^ homeostasis, and impaired contractile function [60]. In response to deficient biogenetics, a failing heart increases heme synthesis for mitochondrial biogenesis, which requires ETC complexes containing iron–sulfur and heme in redox centers [27]. Transgenic mice with ALAS2 (δ-aminolevulinic acid synthase 2), the rate-limiting enzyme in heme synthesis, were characterized by heme accumulation and exacerbation of fibrosis and cell death, while genetic knockdown of ALAS2 in cardiomyocytes reversed heme accumulation and rescued cell viability under hypoxic induction [61]. In a mouse model of hemochromatosis, iron load upregulated oxidative stress, calcineurin-NFAT signaling, fibrosis, and MHC-β expression to exacerbate cardiac hypertrophy [62]. In parallel with chronic hypoxia [8], elevated hematocrit in Ad-hens may promote heme uptake by the heart, leading to cytosolic heme accumulation, HO-1 upregulation, and, thereby, iron release, while heme synthesis and iron-related gene expressions were upregulated for mitochondrial biogenesis. As the remodeling progressed into the failing state after 35 weeks, downregulated iron-related gene expressions disrupted cardiac labile iron disposal, and thus provoked ROS production, leading to cell ferroptosis. Ferroptosis is characterized by lipid peroxidation and ROS over-production due to the Fenton reaction by iron overload and inactivation of GPX4 [18,28]. Treatments with various antioxidants, iron chelators, and other compounds have been reported to suppress ferroptotic cell death and ameliorate heart failure [63,64]. Several genes related to iron transport and disposal, such as FPN, FTH1, HEPH (Hephaestin), and heme catabolism, and biosynthesis including HO-1, biliverdin reductases, heme transporter HRG-1, and FECH, are classified as Nrf-2 target genes [65]. In accordance with those in other tissues [66,67], supplemental 25-OH-D3 is concluded to alleviate cardiac ferroptosis and pathological remodeling through Nrf-2 regulations in heme and iron metabolism for redox homeostasis.

The present results confirmed that supplemental 25-OH-D3 improved the livability of obese hens by alleviating cardiac remodeling and functional failure. The effects were mediated through relieved BNP and MHC-β upregulations, inflammation, and fibrosis to lessen myocardial pathologies, as well as by improving energy status via ETC complex activity, and differentially upregulated PGC-1α, PINKI, and Parkin to sustain mitochondrial biogenesis and dynamics. Supplemental 25-OH-D3 further potentiated cardiac metabolic flexibility in glucose oxidation and pyruvate/lactate interconversion, β-oxidation, and IDH2, αKGDH, and SDH activity to sustain the anaplerotic TCA cycle and limit TG and ceramide accumulation in lipotoxic development. Furthermore, 25-OH-D3 sustained Nrf2 upregulation, relieved oxidative stress and GSH depletion, eased blood hematocrit, and differentially regulated HO-1, FECH, FTH1, and HEPH expressions in heme and iron metabolism to maintain redox homeostasis and cell viability.

## 5. Conclusions

25-OH-D3 ameliorates the cardiac functional compromise to improve the livability of obese hens by relieving myocardial pathological remodeling, potentiating cardiac metabolic adaptions and mitochondrial function to sustain energetic supply, and by operating as antioxidant defense, and heme and iron metabolism, to maintain redox homeostasis and cell survival.

## Data Availability

All the data sets in the present study are available from the corresponding authors upon reasonable request.

## Supplementary Materials

The following supporting information can be downloaded at: https://www.mdpi.com/article/10.3390/antiox13111426/s1, Figure S1: Effects of dietary 25-hydroxycholecalciferol (25-OH-D3) supplementation on bodyweight, feed intake, and mortality of broiler breeder hens provided with restricted (R) or ad libitum (Ad) feed intake; Figure S2: Effects of dietary 25-hydroxycholecalciferol (25-OH-D3) supplementation on obesity development of broiler breeder hens provided with restricted (R) or ad libitum (Ad) feed intake; Figure S3: Effects of dietary 25-hydroxycholecalciferol (25-OH-D3) supplementation on cardiac fibrosis and inflammation of broiler breeder hens provided with restricted (R) or ad libitum (Ad) feed intake; Figure S4. Effects of dietary 25-hydroxycholecalciferol (25-OH-D3) supplementation on cardiac fibrosis and inflammation of broiler breeder hens provided with restricted (R) or ad libitum (Ad) feed intake; Table S1: Primers for quantitative real-time polymerase chain reaction (qRT-PCR) analysis; Table S2: Incidence of cardiac pathological hypertrophy of broiler breeder hens experiencing sudden death; Table S3: Effects of dietary supplementation of 25-hydroxycholecalciferol (25-OH-D3) on the incidence of cardiac hypertrophy of broiler breeder hens provided with restricted or ad libitum feed intake.
