# Supplementary material for: 25-Hydroxycholecalciferol Improves Cardiac Metabolic Adaption, Mitochondrial Biogenetics, and Redox Status to Ameliorate Pathological Remodeling and Functional Failure in Obese Chickens
Source: Antioxidants (Basel). 2024 Nov 20;13(11):1426. doi: 10.3390/antiox13111426 (PMC11590958; doi:10.3390/antiox13111426)
Supplement: Supplementary file 1 [file antioxidants-13-01426-s001.zip › antioxidants-3208517-supplementary.pdf]

Supplementary results

Body Weight and Feed Intake

Release for Ad-feed intake caused a burst of feed consumption in the first week from the prescribed 145 to 210 g/day/hen. Feed intake then declined gradually to 40-41 weeks, and thereafter increased slowly to 155 g/day/hen at age of 45 weeks (Figure S1 panel A). The BW of Ad-hens increased sharply to reach 4.4 kg/hen at age of 36 weeks, declined slightly to 40 weeks, and subsequently increased to ~4.5 kg/hen at 45 weeks, while R-hens with the recommended feed rations exhibited a slow increase of BW from ~3.1 kg to ~3.5 kg (Figure S1 panel B). In contrast to their surviving counterparts, Ad-hens experiencing SD showed a decline of feed intake after 34 weeks with a deficit 40-50 g/day/hen week 38-41, and then a slow increase to reach a deficit 25-40 g/day/hen at the study end at week 45. The feed intake of SD-hens of R groups diverged later at 38-39 weeks and reached 10-15 g/day/hen deficit at the end.

Mortality and Cardiac Pathological Hypertrophy in Hens Experiencing SD

Hens of Ad- and R-groups started to suffered SD at 28 and 32 weeks, respectively (Figure S1 panel C). In consistence with our previous reports [10, 11], supplemental 25-OH-D3 improved livability with mortality being 8.9%, 6.7%, 48.9%, and 31.1% in R, R+25-OH-D3, Ad, and Ad+25-OH-D3 group, respectively ($p=0.05$ in Chi square analysis, Figure S1 panel C). Cardiomyopathies were found in most of SD-birds, as shown by pathological hypertrophy, particularly concentric hypertrophy (Table S2,). Regression analysis with BW of SD-hens normalized to the BW of their surviving counterparts at the same age (BW ratio) showed a progressive decline with the ratio; within Ad-hens the ratio dropped below 1 around 34 to 38 weeks while the ratio remained above 1 in the R-hen group up to 45 weeks (Figure S1 panel D).

The absolute and relative heart weight of SD-hens increased progressively ranging from 17.5-32.5 g/heart and 0.4-0.85% (of 100g BW) in Ad groups, respectively, and 16-20 g/heart and 0.45-0.5% in R groups (Figure S2 panel A). The SD-hens of Ad groups also exhibited a decline of adiposity along the time course from 5.3% to 2.8% (Figure S3 panel A). The values of absolute and relative heart and abdominal fat weight are much higher than those of corresponding surviving counterparts necropsied at 28, 33, or 45 weeks (Figure S2 and S3 panel A and B). These results confirmed that broiler hens with better feed efficiency and propensity of obesity, as indicated by greater BW ratios and adiposity in early ages than their surviving counterparts, are most susceptible to cardiac functional compromise and thus suffer earlier SD due to irreversibly pathological hypertrophy even under R feed intake. Supplemental 25-OH-D3 limited the pathological remodeling and rescued hens' livability.

Table S1. Primers for quantitative real time polymerase chain reaction (qRT-PCR) analysis

Gene	Access number	Primer (5′-3′)	Amplicon (bps)
**GAPDH (Glyceraldehyde-3-phosphate dehydrogenase)**	NM_204305	F: ACAGCAACCGTGTTGTGGAC<br>R: CAACAAAGGGTCCTGCTTCC	98
**BNP (Brain natriuretic peptide, or Natriuretic peptide precursor B; NPPB)**	XM_040651099	F: TCAGCCCTGCCAAAGAAC<br>R: CCGAAGCAGCCAGAATCTCTC	271
**MHC-β (β-cardiac myosin heavy chain, or MHC-7)**	XM_001231408	F: GGTGGACAAGCTGCAGATGAA<br>R: CCAAATTCCGCAAGATCCAG	100
**ND1 (NADH ubiquinone oxidoreductase chain 1)**	MT800504	F: ACCCTAGCCATCATCCTGTT<br>R: TCCTGAGACTAGCTCTGACT	178
**CYTB (cytochrome b)**	MT796732	F: TGCCTCATGACCCAAATCCT<br>R: AGTGTGAGGAGGAGGATTACT	251
**COX1 (Cytochrome oxidase 1)**	MT800504	F: TCCTTCTCCTACTAGCCTCA<br>R: AGGAGTAGTAGGATGGCAGT	264
**TFR (Transferrin receptor)**	NM_205256.2	F: GCCAGGTGTCAAGATGGAAGTG<br>R: TTGTATCTTCAGCCTCACCAGC	226
**FTH1 (Ferritin, heavy chain 1)**	NM_205086.1	F: ATCGTGATGACTGGGAGAATGG<br>R: CTTGATGGCTTTCACCTGCTC	173
**FPN (Ferroportin-1, or Solute carrier family 40 protein; SLC40A1)**	NM_001012913.1	F: AGACTGGGTGGACAAGAACTCG<br>R: TGCCAGTTTGCTTCTGTCTTCC	274
**HEPH (Hephaestin)**	XM_420165.6	F: GATTCAGCCAACTTATTCCCCC<br>R: CTGGTAATGATTGCTCGTCTGG	102

F: forward, R; reverse

Table S2. Incidence of cardiac pathological hypertrophy of broiler breeder hens experiencing sudden death [1]

	Restriction	Restriction +25-OH-D3	Ad libitum	Ad libitum +25-OH-D3
**Mortality (SD hens/total)**	4/45	3/45	22/45	14/45
**(hearts/SD hens)**				
**Physiological hypertrophy**	0/4	0/3	2/22	1/22
**Concentric hypertrophy**	4/4	3/3	15/22	11/14
**Dilation (eccentric hypertrophy)**	0/4	0/3	5/22	2/14
**Pericardial effusion** [2]	0/4	0/3	3/22	2/22

1. Hens experiencing sudden death (SD) along the time course were recorded and necropsied for body composition measurement (total n=4, 3, 22, 14 for R, R+25-OH-D3, Ad, and Ad+25-OH-D3 group, respectively) and cardiac morphology examination.
2. Two and 1 of dead Ad-hens exhibited pericardial effusion in complication with the presence of concentric hypertrophy and eccentric hypertrophy, respectively. One hen of Ad-25-OH-D3 group showed pericardial effusion in complication with the presence of concentric hypertrophy or eccentric hypertrophy.

Table S3. Effects of dietary supplementation of 25-hydroxycholecalciferol (25-OH-D3) on the incidence of cardiac hypertrophy of broiler breeder hens provided with restricted or ad libitum feed intake

hearts of necropsied hens	Restriction	Restriction +25-OH-D3	Ad libitum	Ad libitum +25-OH-D3
**Incidence of pathological morphology [1] (hearts/total)**	5/18	3/18	15/18	11/18
**at age of 28 wks**				
**Absence of hypertrophy**	4/4	4/4	0/4	0/4
**Physiological hypertrophy**	0/4	0/4	2/4	3/4
**Concentric hypertrophy**	0/4	0/4	2/4	1/4
**at age of 33 wks**				
**Absence of hypertrophy**	4/7	5/7	0/7	0/7
**Physiological hypertrophy**	1/7	1/7	1/7	2/7
**Concentric hypertrophy**	2/7	1/7	5/7	5/7
**Dilation (eccentric hypertrophy)**	0/7	0/7	1/7	0/7
**at age of 45 wks**				
**Absence of hypertrophy**	3/7	4/7	0/7	0/7
**Physiological hypertrophy**	1/7	1/7	0/7	2/7
**Concentric hypertrophy**	3/7	2/7	5/7	4/7
**Dilation (eccentric hypertrophy)**	0/7	0/7	2/7	1/7
**Pericardial effusion [2]**	0/7	0/7	1/7	0/7

1. Surviving hens at age of 28, 33, and 45 weeks were necropsied for body composition analysis and examination of cardiac pathological remodeling (n = 4, 7, 7 from each group).
2. One hen exhibited pericardial effusion in complication with the presence of concentric hypertrophy in Ad group.

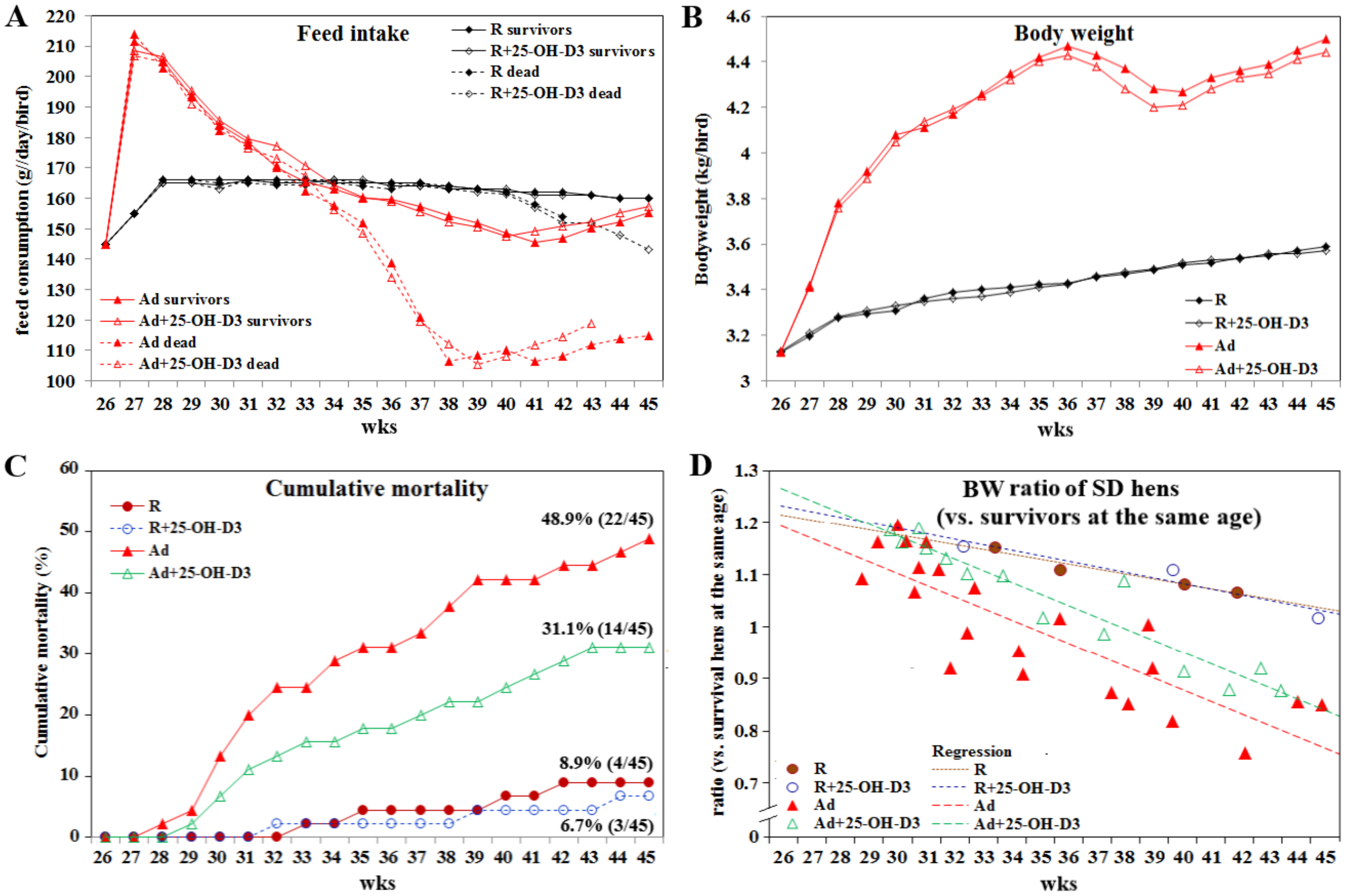

Chi-square (χ2)=29.14, P<0.0001 by Ad

χ2=5.88, P=0.05 by 25-OH-D3

Supplementary Figure S1. Effects of dietary 25-hydroxycholecalciferol (25-OH-D3) supplementation on bodyweight, feed intake, and mortality of broiler breeder hens provided with restricted (R) or ad libitum (Ad) feed intake. At age 26 weeks, 90 birds were maintained with restricted rations (R-hens), while another 90 birds were released for ad libitum feed intake (Ad-hens). Within each feed intake treatment, half of hens consumed a standard diet, while the other half consumed the same diet containing additional 69 μg/kg feed of 25-OH-D3 (panel A). Bodyweight was recorded weekly from 15 birds of each group (panel B). Results of feed intake were separated into those for hens surviving to 45 weeks labeled "survivors"(n = 45 before 28 weeks for each group, n = 40, 40, 30, and 35 during 29–33 weeks, and n = 30, 31, 12, and 20 during 34–45 weeks, for R, R+25-OH-D3, Ad, Ad+25-OH-D3 group, due to 4, 7, 7 hens sampled at 29, 35, and 45 weeks, respectively). Hens experiencing sudden death (SD) were labeled as "dead" along the trial (n = 4, 3, 22, and 14, for R, R+25-OH-D3, Ad, and Ad+25-OH-D3 group respectively). SD-hens along the time course were recorded and necropsied for body composition measurement. The mortality was calculated at the cumulative basis (panel C). The body weight ratio of SD-hens represents the relative value to that of those hens remaining alive within the same group at the same week of age (panel D). Values for individual SD-hens are shown as scattered points. Individual data points from a treatment group were used to calculate the trend line using linear regression.

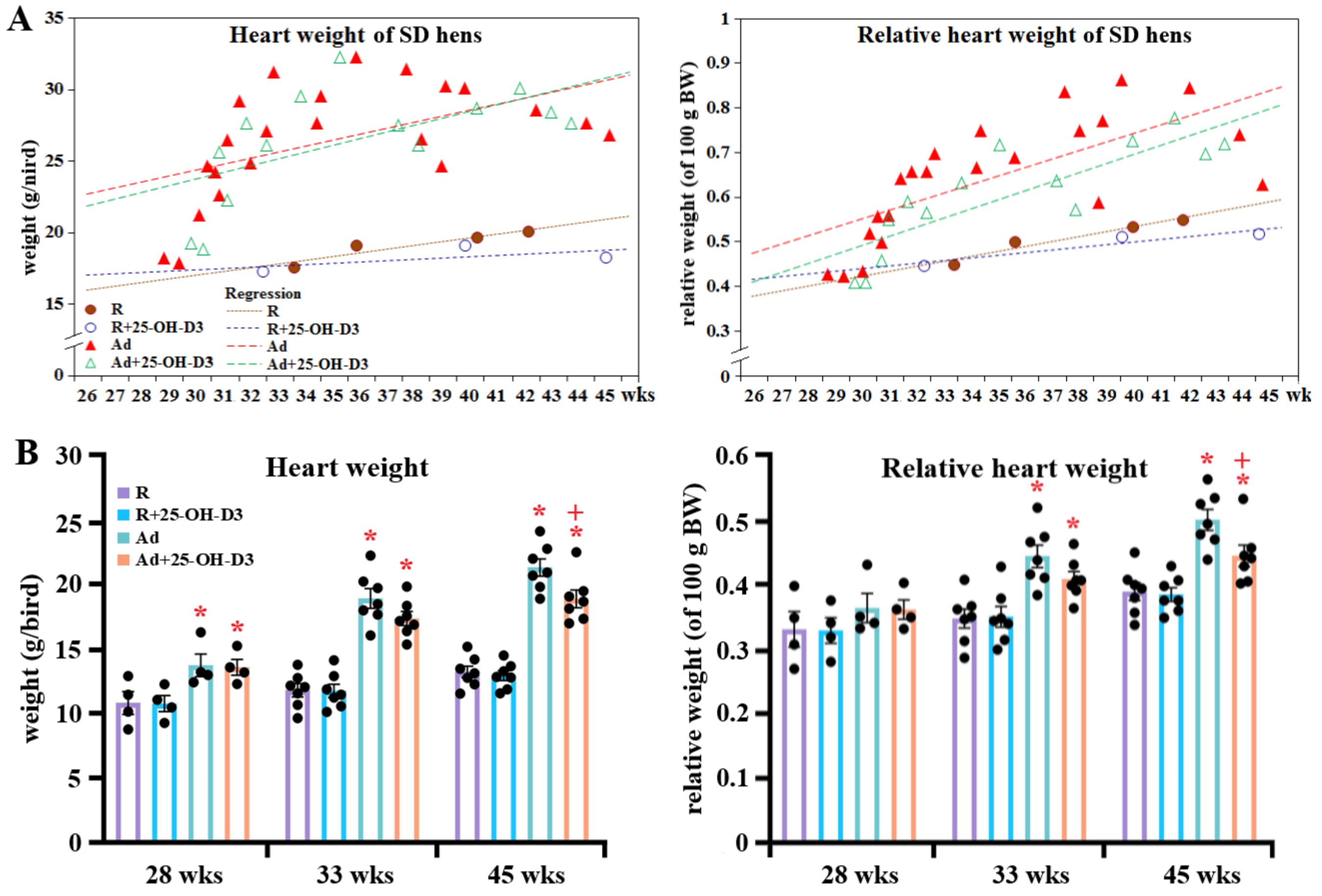

Supplementary Figure S2. Effects of dietary 25-hydroxycholecalciferol (25-OH-D3) supplementation on cardiac hypertrophic growth of broiler breeder hens provided with restricted (R) or ad libitum (Ad) feed intake. At age 26 weeks, 90 birds were maintained with restricted rations (R-hens) as recommended, while another 90 birds were released for ad libitum feed intake (Ad-hens). Within each feed intake treatment, half of hens consumed a standard diet, and the other half consumed the same diet containing additional 69 μg/kg feed of 25-OH-D3. Hens experiencing sudden death (SD) along the time course (panel A; total n = 4, 3, 22, 14 for R, R+25-OH-D3, Ad, and Ad+25-OH-D3 group, respectively) and surviving hens at age of 28, 33, and 45 weeks (panel B; n = 4, 7, 7 from each group) were necropsied for body composition analysis. Heart weight change was evaluated for cardiac hypertrophic growth. *; significant difference by Ad-feed intake (*vs*. corresponding R-hens, $P < 0.05$). +; significant difference by 25-OH-D3 (*vs*. R or Ad hens, $P < 0.05$).

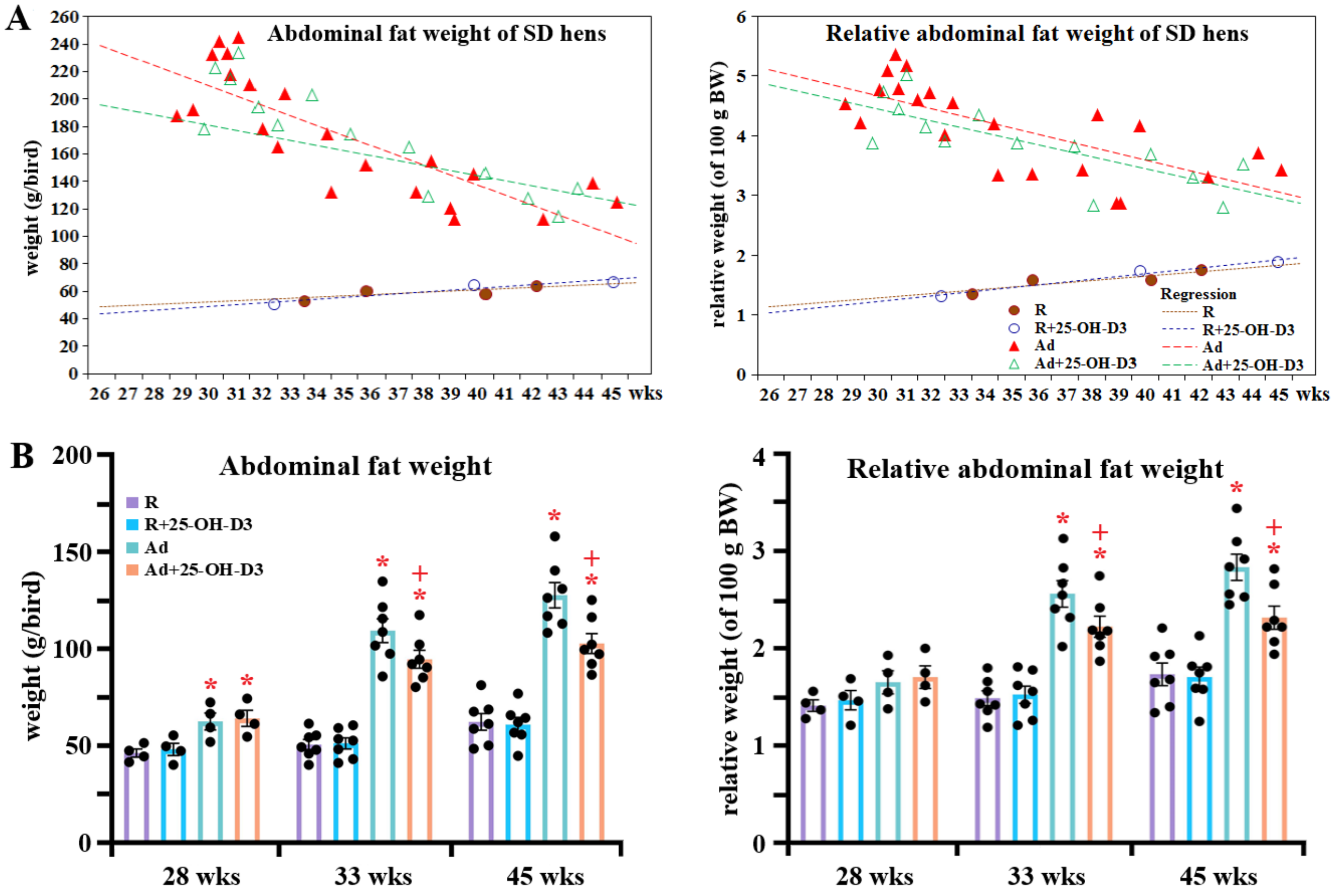

Supplementary Figure S3. Effects of dietary 25-hydroxycholecalciferol (25-OH-D3) supplementation on obesity development of broiler breeder hens provided with restricted (R) or ad libitum (Ad) feed intake. At age 26 weeks, 90 birds were maintained with restricted rations (R-hens) as recommended, while another 90 birds were released for ad libitum feed intake (Ad-hens). Within each feed intake treatment, half of hens consumed a standard diet, and the other half consumed this same diet containing additional 69 μg/kg feed of 25-OH-D3. Hens experiencing sudden death (SD) along the time course (panel A; total n = 4, 3, 22, 14 for R, R+25-OH-D3, Ad, and Ad+25-OH-D3 group, respectively) and surviving hens at age of 28, 33, and 45 weeks (panel B; n = 4, 7, 7 from each group) were necropsied for body composition analysis. Abdominal fat weight change was evaluated for obesity development. *; significant difference by Ad-feed intake (vs. corresponding R-hens, $P < 0.05$). +; significant difference by 25-OH-D3 (vs. R or Ad hens, $P < 0.05$).

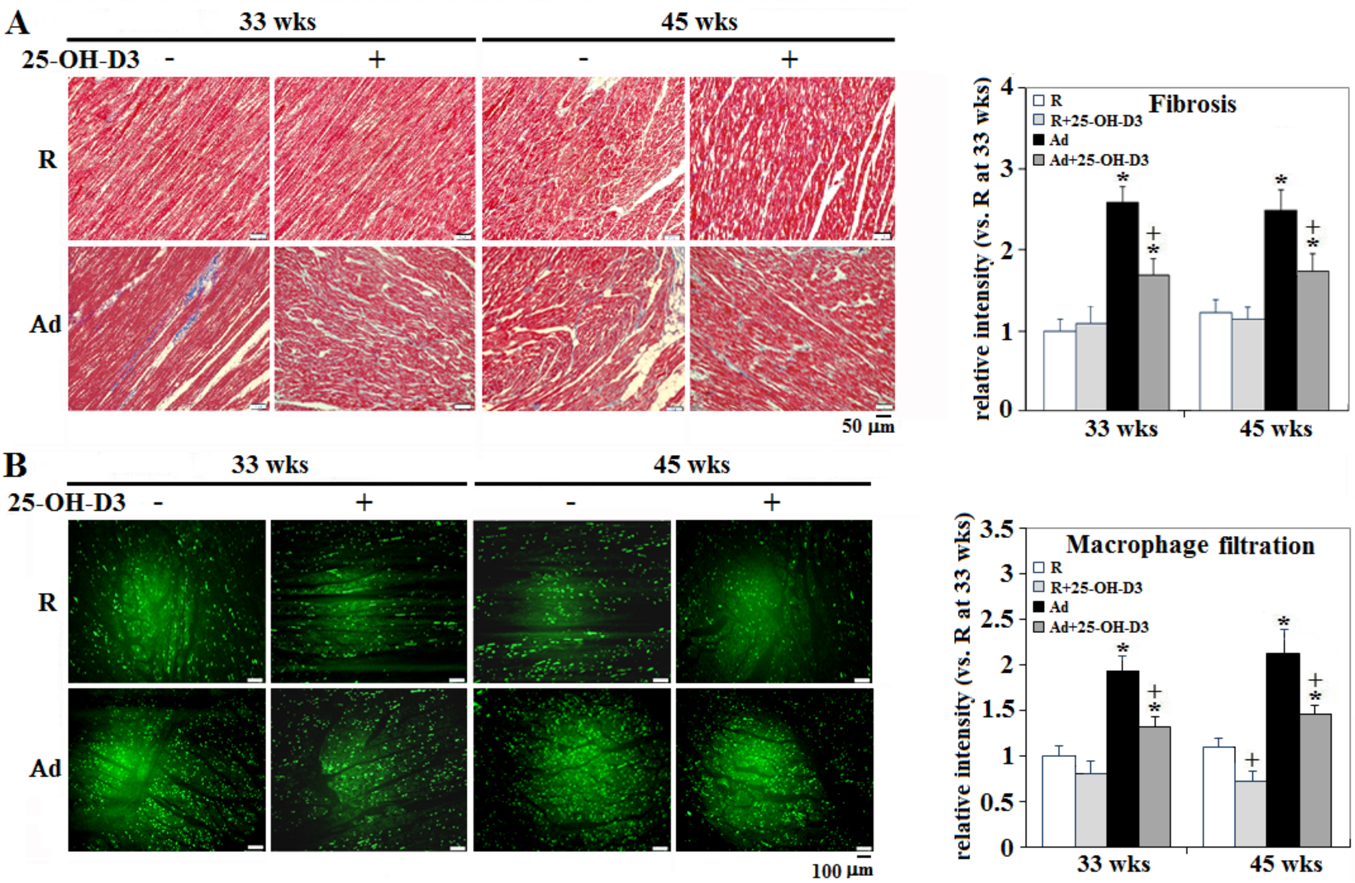

Supplementary Figure S4. Effects of dietary 25-hydroxycholecalciferol (25-OH-D3) supplementation on cardiac fibrosis and inflammation of broiler breeder hens provided with restricted (R) or ad libitum (Ad) feed intake. Living hens at age of 28, 33, and 45 weeks were necropsied for tissue collection (n = 4, 7, 7 from each group). Fibrosis was determined by collagen intensity under trichrome staining (blue color, panel A) and macrophage filtration was determined by immunohistochemistry using a specific antibody against avian macrophages (panel B). Results were expressed as ratios relative R-hens at 33 weeks (n = 3). *; significant difference by Ad-feed intake (*vs*. R-hens at 33 weeks, $P < 0.05$). +; significant difference by 25-OH-D3 (*vs*. R- or Ad-hens, $P < 0.05$).
